# Preparative Coupled Enzymatic Synthesis of L-Homophenylalanine and 2-Hydroxy-5-oxoproline with Direct In Situ Product Crystallization and Cyclization

**DOI:** 10.1021/acsomega.5c00590

**Published:** 2025-04-02

**Authors:** Sven Tiedemann, Annabel Stang, Simon Last, Thierry Gefflaut, Jan von Langermann

**Affiliations:** † Otto von Guericke University Magdeburg, Institute of Chemistry, Biocatalysis Group, Universitätsplatz 2, 39106 Magdeburg, Germany; ‡ Université Clermont Auvergne, Institut de Chimie de Clermont-Ferrand, 24 avenue des Landais, 63178 Aubiere Cedex, France

## Abstract

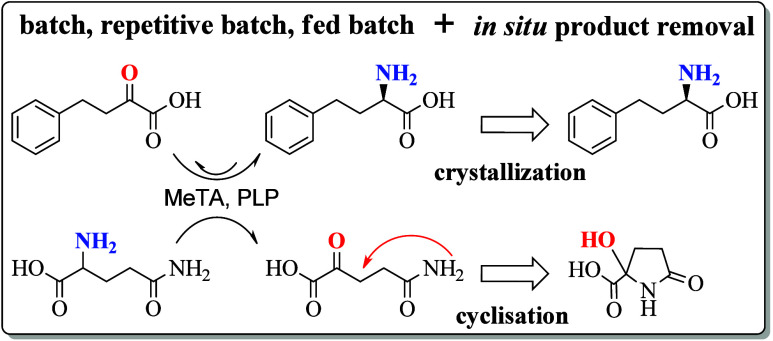

A continuous in situ crystallization concept is presented for the coupled preparative synthesis of L-homophenylalanine and 2-hydroxy-5-oxoproline (a cyclized form of α-ketoglutarate) using the α-transaminase from *Megasphaera elsdenii*. The process consists of a spontaneous reactive crystallization step of the enantiopure amino acid itself and a parallel spontaneous cyclization of the deaminated cosubstrate in solution. In parallel, these effects significantly improve the overall productivity of the biocatalytic reaction. Batch, repetitive, and fed-batch processes were investigated, and the fed-batch option proved to be the most viable option. The fed-batch process was subsequently used for a coupled synthesis approach at the gram scale. In total, >18 g of chemically pure L-homophenylalanine and >9 g of 2-hydroxy-5-oxoproline were isolated. This optimized process allows for the design of effective transaminase-catalyzed reactions at a preparative scale utilizing standard (fed-)batch-mode crystallizers.

## Introduction

The use of biocatalysis as a powerful alternative to classical chemical reaction systems has increased in recent decades due to their ability to produce highly valuable, often enantiopure products.^1−4^ A major class of biocatalytically derived product groups are chiral amines, which serve as important intermediates in the pharmaceutical and agro-industrial fields.^5,6^ Transaminases (TAs) are particularly important, as they are extremely efficient biocatalysts for the synthesis of chiral amines and amino acids using only a donor amine and the inexpensive cofactor pyridoxal phosphate.^7,8^ However, within preparative applications, transaminase reaction systems often encounter unfavorable reaction equilibria in asymmetric synthesis.^9^ Especially, at higher reactant loadings, substrate and/or product inhibition occurs, which must be overcome to achieve efficient process conditions.^10,11^

**Scheme 1:**
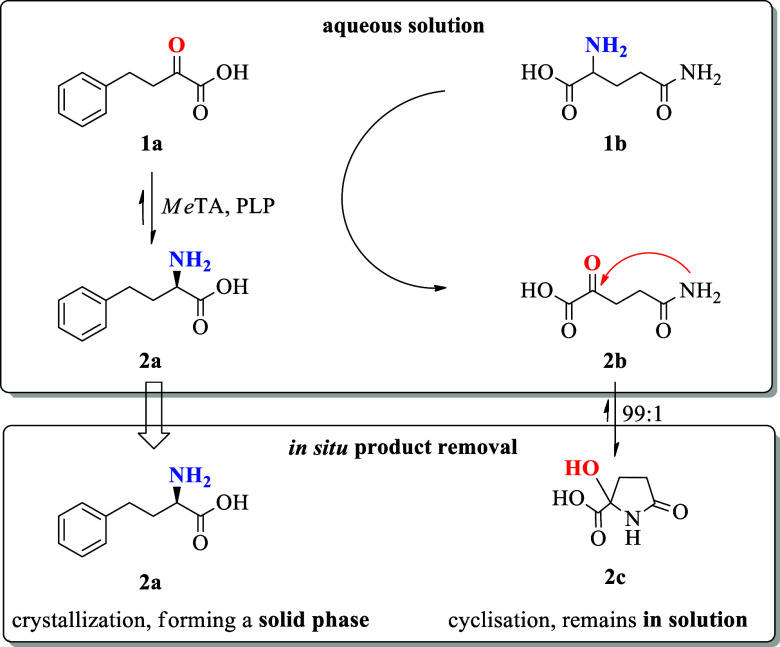
General Reaction Concept, Involving the Transaminase from *Megaphaera elsdenii* (*Me*TA)-Catalyzed Conversion of OPBA (**1a**) to L-HPA (**2a**) with l-Glutamine (**1b**) Deamination to α-Ketoglutaramat (αKGM, **2b**), which Spontaneously Cyclizes to 2-Hydroxy-5-oxoproline (HOP, **2c**)

The most straightforward approach is the removal of one or more (co)products from the reaction using in situ product removal, e.g., through evaporation or specifically designed cascades.^12−14^ This study focuses on the use of in situ-product crystallization, which has recently proved its general applicability on a preparative scale.^12,15,16^ This concept involves transferring one or more reactants of the (bio)chemical reaction equilibrium from within the aqueous solution into one or more solid phases to reduce the occurrence of product inhibition. This process itself and specifically the accumulation of (co)products is entirely controlled by the solubility limit of the product or its respective sparingly soluble salt.^17^ Thus, the process is intrinsically dependent on the (low) solubility of the products and required crystallization for general usage. Moreover, in situ product crystallization can simplify downstream processing as it is significantly easier to remove the product through filtration therefore reducing effort and the use of auxiliary chemicals.^9,18,19^ Additionally, it can be used to create solutions to obtain enantiopure products from a racemic mixture.^20^ Previous studies have specifically applied sterically demanding organic carbonic acids or the corresponding carboxylates, which allows for the targeted crystallization of chiral amines as its sparingly soluble ammonium salt.^17,21^ Although preparative use is feasible with this technique, direct reactive crystallization is preferred as no crystallization-based additives are required. An example that has gained interest in recent years is the non-natural amino acid L-homophenylalanine (HPA), which serves as an intermediate for the synthesis of ramipril, enalapril, imidapril, and others (Scheme 1).^22−27^ The biocatalytic process itself is considered significantly simpler when compared to the original multistep synthesis.^28−33^ This is usually done via transaminase pathways, but can also be achieved with dehydrogenases and β-decarboxylase.^7,27,34,35^ However, the remaining issue is often the low atom efficiency due to side-products, which need to be overcome for this specific enzyme-catalyzed reaction.^36−38^ One potential strategy to overcome this limitation is the design of combined pathways that facilitate the conversion of all substrates into usable products, e.g., a coupled synthesis in which both product and coproduct are valuable.^5,39,40^ This is particularly advantageous if the separation and purification processes for both components are simple and the remaining impurities from the biocatalysts step (protein, cell debris, etc.) can be easily removed.^41^

This study presents the implementation of reactive crystallization of HPA using an α-transaminase at a preparative scale, starting from 2-oxo-4-phenylbutanoic acid (OPBA), as a one-step-reaction concept under optimized conditions. This includes optimizing the enzyme by implementing a developed reactor setup to reduce the cost of the process.^24,42−44^ The driving force of this reaction is that the product, HPA, is only sparingly soluble in aqueous systems between pH 2 and 10. This naturally leads to a direct crystallization of the product from the biocatalytic reactions mixture.^35,45^ Within the α-transaminase-catalyzed reaction l-glutamine is applied as a smart amine donor as it results in a spontaneous equilibrium displacement of the deaminated coproduct α-ketoglutaramate (αKGM) via a spontaneous cyclization to 2-hydroxy-5-oxoproline (HOP).^7,46−48^ HOP can be produced in multiple ways such as vanadium oxide catalysts and l-glutamate oxidase.^49−51^ Interestingly, this coproduct is also currently being investigated as a potential substrate for ω-amidases in pharmaceutical research.^47,52^ While HPA is directly obtained as a solid, HOP is isolated independently via a separate ion-exchanger-based downstream-processing approach, yielding both products in a coupled synthesis at the preparative scale.

## Materials and Methods

### Biocatalyst and Chemicals

All biocatalysts were prepared through overexpression in **E. coli** BL21 cells and used either as cell-free extract or whole cells (recombinant in **E. coli**). All samples were used as lyophilizate. Further details about enzyme origin and preparation can be found in ref (7). 2-Oxo-4-phenylbutanoic acid 98% was bought from BLDpharm (Shanghai, China), l-glutamine >99% from TCI (Portland, USA), DL-homophenylalanine 98% from Sigma-Aldrich (St. Louis, USA), L-homophenylalanine 98% from Carbolution (St. Ingbert, Germany), pyridoxal phosphate 98% from fluorochem (Hadfield, U.K.), and Dowex 50WX8 from Carl Roth (Karlsruhe, Germany).

### Enzyme Activity Assay

Enzyme activity was measured via a conversion assay with the help of a comparison reaction system. 25 mg of biocatalyst was added to a 1 mL solution containing 50 mM (8.9 mg) OPBA, 60 mM (8.8 mg) Gln, and 5 mM (1.3 mg) PLP. After 30 min at 750 rpm and 30 °C, the reaction was terminated with the addition of conc. HCl solution (50 μL). A sample was taken according to the sample analysis step, and the activity relative to the weight was calculated based on the produced L-HPA. One unit (U) is defined as the conversion of 1 μmol of OPBA toward HPA within 1 min. An average expression resulted in ∼3.0 U/mg lyophilized cell lysate/whole cells.

### Solubility Tests

In an 8 mL Vial, 200 mg of substrate (**1a**) and 5 mL of 50 mM pH 8 phosphate buffer solution were combined, and vials were shaken horizontally. After a few days, the pH was adjusted and the vials were shaken again until the pH did not change, for a minimum of 7 days to ensure equilibrium conditions. Afterward, 1 mL of the solution was filtered through a sterile filter (0.22 μm) into a smaller glass vial to remove any undissolved components. The solution was evaporated until it was dry and solubility was calculated based on the weight of the remaining solid.

### Single Batch Reactor

In a 2 mL test tube, 100 mM (17.8 mg) of OPBA and 120 mM (17.5 mg) l-Gln were weighed. 1 mL of 50 mM phosphate buffer pH 8 containing 5 mM PLP was added. After correction of the pH to the desired value, 100 U/mL of catalyst (whole cells or crude extract) was added to start the reaction.

### Repetitive Batch Reaction

A 10 mL batch reactor with 100 mM (178 mg) **1a**, 120 mM (175 mg) **1b,** and 5 mM (12 mg) PLP was initiated with a catalyst in the form of a crude extract and operated for 8 h. A 500 μL sample was taken for HPLC analysis after the reaction had finished and treated as mentioned below (Sample analysis) but with the respective volumes halved. The remaining contents were transferred to a 50 mL falcon tube and centrifuged at 4000 rpm for 15 min, and the supernatant was collected using a syringe. A stock solution of 50 mM phosphate buffer pH 8, containing 5 mM PLP, was used to readjust to a 10 mL reaction volume. The reactor was then restarted with the addition of a substrate, donor amine, as well as 10% additional enzyme and run for 16 h (overnight). The entire process was then repeated (3–5 days).

### Fed-Batch Reaction

In a 500 mL flask, 30 mM (0.53 g) OPBA and 34.5 mM Gln (0.5 g) were prepared with 100 mL of 50 mM phosphate buffer pH 8 and 5 mM (36 mg) PLP. To this mixture, 3 g of enzyme (whole cells, 3.28 U/mg = 100 U/mL) was added, and the reaction was stirred at 700 rpm in a 40 °C oil bath. In a second flask, 280 mL of the same buffer solution containing 400 mM (19.96 g) **1a** and 480 mM (18.82 g) 1b are prepared. Through a pump 10 mL/h, this reservoir was added to the reaction over a 24 h time frame.

### Sample Analysis

To 1 mL of reaction sample, 50 μL conc. HCl was added. The mixture was vortexed for 30 s and 250 μL was transferred to 1 mL of a 1:1 mixture of methanol and acetonitrile. This was vortexed for 30 s and then centrifuged for 5 min to remove any residual enzymes and undissolved reactants. From this, 525 μL was added to 975 μL 10 mM phosphate buffer pH 4. This sample can be further diluted by adding additional solvents to adjust the final concentrations.

### HPLC Analysis

A Shimadzu HPLC system (Ort, Land, SCL-40, DGU-405, LC-40D, SIL-40C, CTO-40S, SPD-M40) was used with a Kinetex 2.6 μm C18 100 Å″ Column of 150 mm × 3 mm size. The mobile phase consisted of a 35% mixture of 1:1 methanol and acetonitrile with 65% 10 mM phosphate buffer pH 4. Samples were analyzed at 0.12 mL/min at 40 °C and a runtime of 15 min at a wavelength of 245 nm. Signals of **2a** (7 min) and **1a** (10 min) were used for calculations.

### Determination of Enantiomeric Excess

A Shimadzu HPLC system (Ort, Land, SCL-40, DGU-405, LC-40D, SIL-40C, CTO-40S, SPD-M40) was used with “Chirex 3126 (D)-penicillamine” at 150 × 4.6 mm. The mobile phase consisted of 85% 2 mM CuSO_4_ in water and 15% acetonitrile. Samples were analyzed at 1 mL/min at 40 °C and a runtime of 60 min at a wavelength of 245 nm. Retention times: 27 min L-HPA (**2a**) and 30 min D-HPA.

### Product **2a** Isolation

The final reaction suspension was transferred into 50 mL centrifuge tubes and centrifuged at 4000 rpm for 20 min at 4 °C. The resulting solid-free solution was stored independently for subsequent coproduct extraction. To the remaining solids, 10 mL of concentrated hydrochloric acid was added per ca. 1 g of raw product. The tubes were shaken well to dissolve the entire product and subsequently centrifuged again for 20 min (4000 rpm, 4 °C, to remove all remaining cell residues). All solutions were combined, and the pH was adjusted to 7.5 by the addition of a saturated sodium hydroxide solution. The mixture was placed at 4 °C overnight, and the resulting solid **2a** was isolated by filtration, washed with cold water, and dried under reduced pressure.

### Recrystallization of L-homophenylalanine (for XRPD Analysis)

Option A: L-homophenylalanine (100 mg) was placed in a flask with an additional glass construction on top. Vacuum was applied, and the solid was heated to 175 °C. After 2 days, pure crystalline needles formed on the sides of the flask as well as on a needle placed in the middle of the structure.

Option B: L-homophenylalanine (200 mg) was dissolved in a flask containing a 25 mL mixture of concentrated hydrochloric acid and distilled water (roughly 1:2; pH < 1). The solvent was slowly evaporated, resulting in crystalline needles.

### Product **2c** Isolation

The solid-free solution from **2a** isolation was collected in a round-bottom flask, and its initial volume (ca. 300 mL) was reduced under vacuum until about 10 mL remained. The solution was filtered and poured over a conditioned Dowex 50WX8 column (20 mL), which was cleaned and prepared with hydrochloric acid beforehand. Fractions containing **2c** were collected, reduced, and poured over a silica gel column (50 mL), to remove any remaining impurities, and reduced again. This resulted in 9.6 g (49.7%) of an orange highly viscous liquid which can be stored in a fridge for approximately 1 week. To prolong storage time, a lithium salt of **2c** can be prepared by the addition of LiOH and lyophilization.^7,53^

### Determination of Potential Protein Impurities

200 mg of the product was added to a 500 μL solution of 50 mM phosphate buffer at pH 8, and the mixture was shaken for 30 min. Following this, the mixture was centrifuged, 3 × 10 μL was transferred to a 96-well plate, and 200 μL of Bradford solution was added to each. After a 5 min incubation period, the samples were measured at 595 nm against a blank to determine the remaining protein content (see SI).

## Results and Discussion

### Optimization of the Enzymatic HPA Synthesis

**Figure 1:**
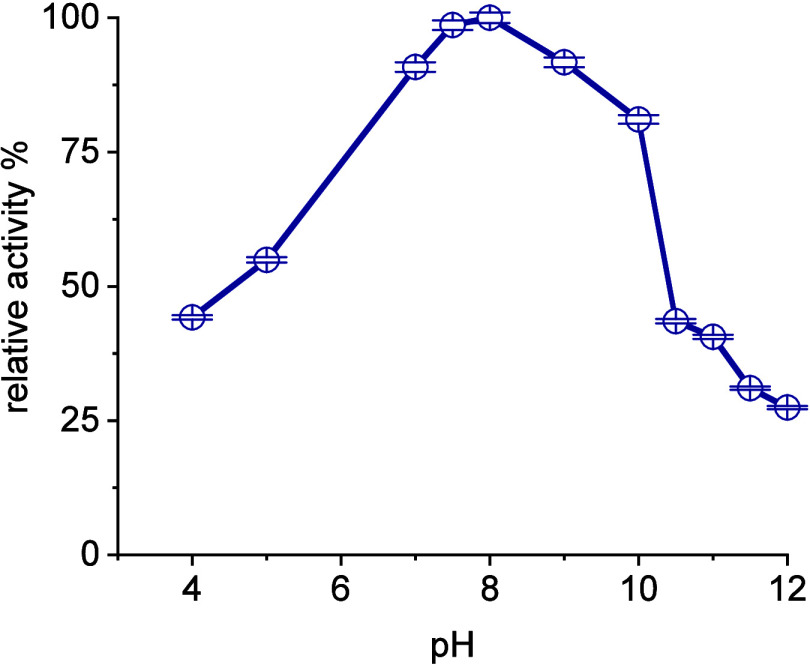
pH-dependency of *Me*TA; Reaction conditions: 100 mM **1a**, 120 mM **1b**, 5 mM PLP, 50 mM phosphate-buffer, 30 °C, 24 h, 100 U/mL catalyst.

Initial investigations targeted the fundamental understanding of the underlying reaction system and its simultaneous crystallization. The solubility of substrate **1a** is especially relevant, as its availability in the aqueous reaction is crucial for all subsequent reaction steps. Solubility experiments of **1a** unfortunately led to highly viscous media, which made it challenging to accurately measure solubility across a wide range of pH values. However, it was determined that a minimum of 0.1 g/mL will be soluble most of the time. It is worth noting that **1a** may require a prolonged time to reach solubility equilibrium. Furthermore, it was observed that **1a** dissolves slowly but consistently, taking on a slight yellow appearance under the influence of basic environments. Fortunately, amine donor **1b** as the secondary substrate is a conventional L-amino acid that has been extensively characterized, e.g., Heuson et al. in 2019 for the conversion of 2-oxo-phenylbutyric acid to L-homophenylalanine. Both products **2a** and **2b** undergo a spontaneous transformation that results in in situ-product removal and thus removal from the underlying reaction equilibrium. First, **2a** exhibits a very low solubility and spontaneously crystallizes as a white powder. Second, **2b** is independently cyclized to form **2c** at a ratio of at least 1:99, which further supports the shift of the reaction equilibrium of the product side toward **2a**. Both effects basically circumvent the possibility of substrate inhibition caused by these compounds, creating an opportunity for scaling up the synthesis after process optimization. At the beginning of the investigations, the pH-dependent behavior was examined (Figure 1) and the optimal conditions for the enzyme were found to be pH 8 ± 0.5, which is positioned within the typical range of many transaminases. The behavior at higher pH is also of interest, as **2a** becomes more soluble above pH 10, thus enabling other process options. This opens up the possibility of performing the reaction classically without any form of in situ-product crystallization at higher pH and a subsequent controlled crystallization using a split reactor and crystallizer setup.

**Figure 2:**
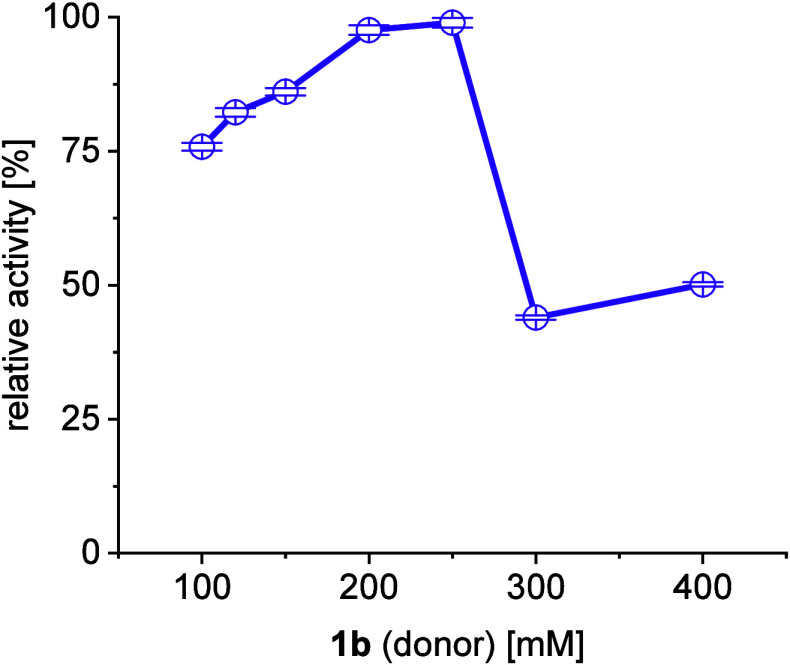
Investigation of the behavior of *Me*TA toward higher donor (**1b**) concentrations with a 1 mL batch reaction of 100 mM **1a**, 5 mM PLP, 50 mM phosphate-buffer pH 8, 30 °C, 24 h, 100 U/mL catalyst.

**Figure 3:**
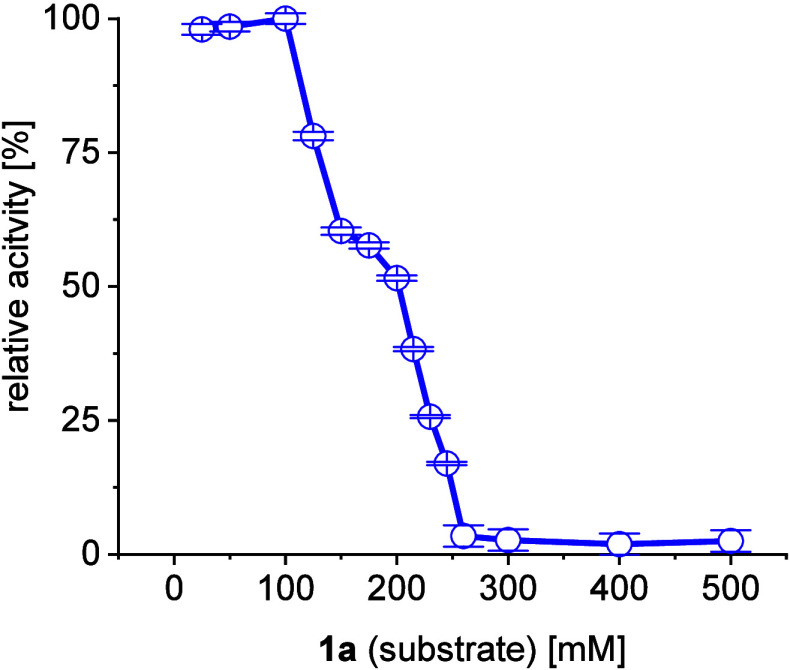
Investigation of substrate (**1a**, **1b**) inhibition for *Me*TA with a 1 mL batch reaction, C_1a_:C_1b_ (1:1.2), 5 mM PLP, 50 mM phosphate-buffer pH 8, 30 °C, 24 h, 100 U/mL catalyst.

**Figure 4:**
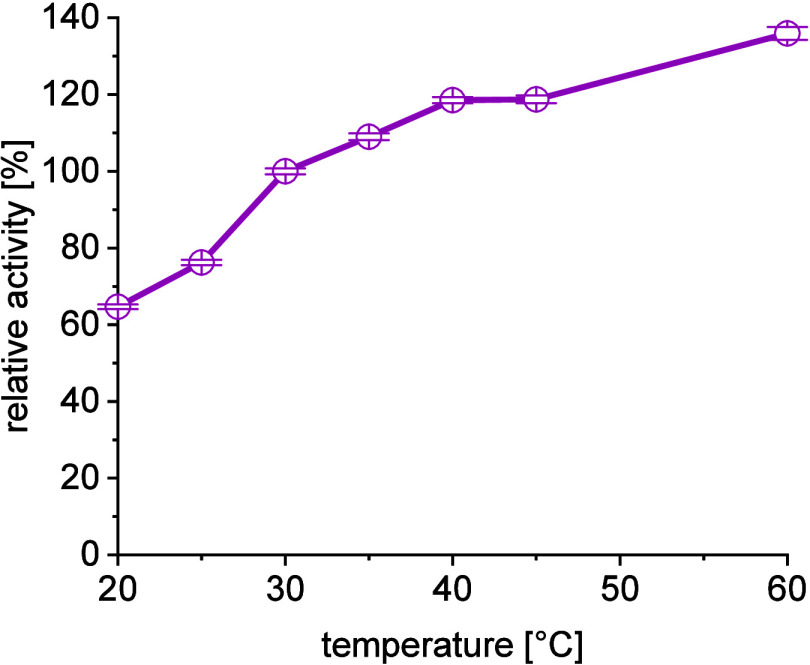
Temperature dependency of *Me*TA for a 1 mL batch reaction of 100 mM **1a**, 120 mM **1b**, 5 mM PLP, 50 mM phosphate-buffer pH 8, 24 h, 75 U/mL catalyst.

Furthermore, substrate inhibition was investigated in detail to support process optimization. Figure 2 shows that higher amounts of the donor (**1b**) will improve conversion, as expected, but only up to a 250 mM threshold. However, a 1:1.2 ratio of **1a**/**1b** was already sufficient for reaching conversions of over 95%, highlighting the fact that no direct benefit is obtained in raising the amount of the donor to such excessive nonstoichiometric amounts. Moreover, substrate inhibition based on substrate **1a** occurs similarly at concentrations above 100 mM and leads to a rapid decline in the conversion rate (Figure 3). The reaction system even came to a complete halt when the substrate concentration was raised beyond 250 mM. Therefore, it is preferred to run the reaction at a constant concentration of 100 mM or below **1a** to maintain sufficient reactivity. Figure 4 shows the temperature dependency of the reaction system with slightly reduced enzyme addition to compensate for higher reactivities at elevated temperatures as the applied standard setup was already approaching full conversions at 30 °C. As expected, increasing the reaction temperature generally increased overall conversion (after 24 h). A decrease in enzyme stability needs to be taken into account at higher temperatures, and thus 40 °C was chosen as a compromise throughout all subsequent investigations. It should be noted that further improvements in enzyme stability are theoretically possible via enzyme immobilization, but were not investigated in detail here, as any kind of secondary solid phase, besides crystallized product **2a**, should be avoided.

**Figure 5:**
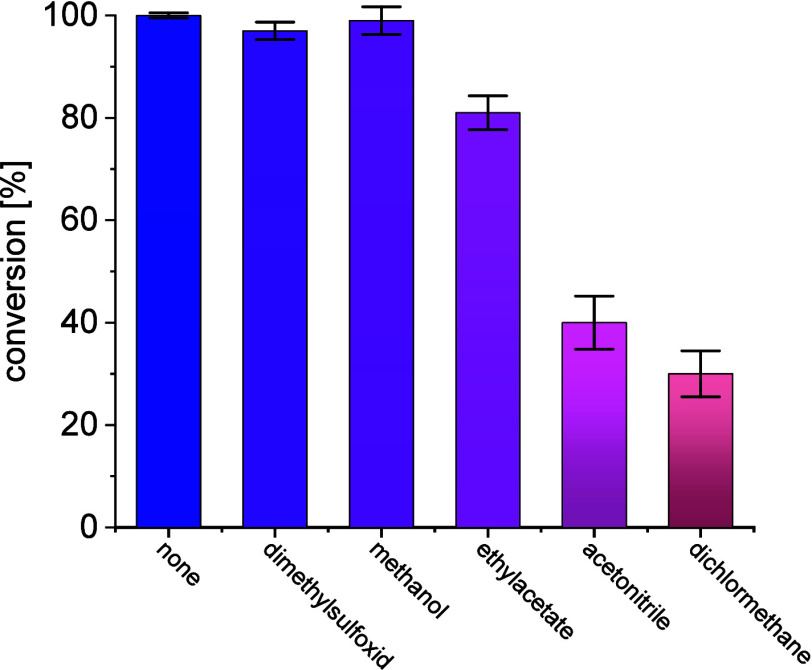
Effect of organic solvents on the performance of *Me*TA, 100 mM **1a**, 120 mM **1b**, 5 mM PLP, 50 mM phosphate-buffer pH 8, 40 °C with addition of 20 vol % organic, 100 U/mL catalyst.

Moreover, the tolerance of the catalyst toward the addition of organic solvents was recorded as such solvent additions are often considered in transaminase-catalyzed reaction systems (Figure 5). Some cosolvents do not appear to inhibit the reaction at all at low concentrations of 20 v/v% or less (dimethyl sulfoxide, methanol), while others harshly reduce the performance like acetonitrile and dichloromethane. As no positive effect was observed while using cosolvents, their use was not considered relevant.

**Figure 6:**
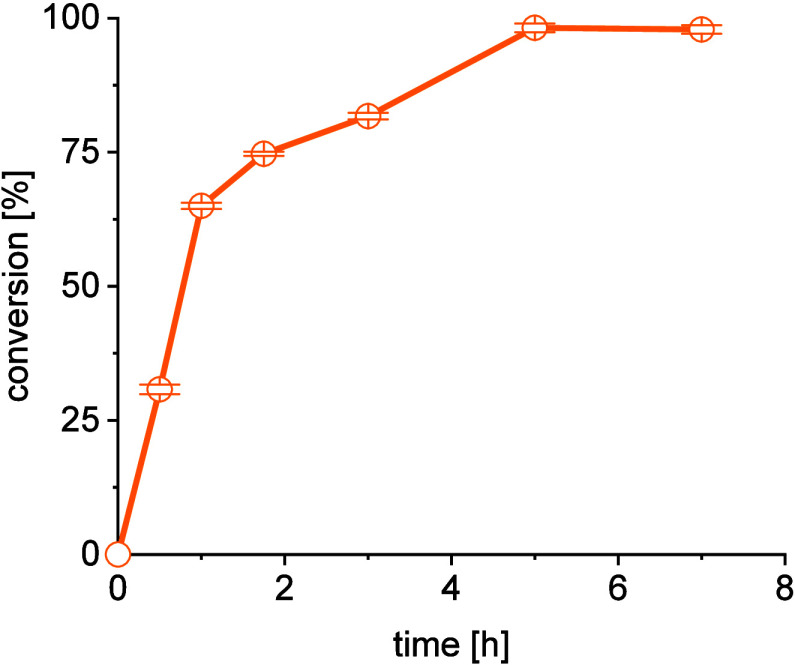
Time-dependent conversion for a reaction of 100 mM **1a**, 120 mM **1b**, 5 mM PLP, 50 mM phosphate buffer, pH 8, 40 °C, 100 U/mL catalyst.

Under optimized reaction conditions, full conversion toward **2a** was achieved within 5 h, which is considerably faster than many other reported transaminase-catalyzed reaction systems (Figure 6). This results from the double equilibrium shift in favor of the products (crystallization of **2a** and cyclization of **2b** to **2c**), which significantly increases the reaction rate. Both effects also enable the very simple use of more complex reaction concepts, such as repetitive batch and fed-batch systems (see below).

### Repetitive Batch Reaction

**Figure 7:**
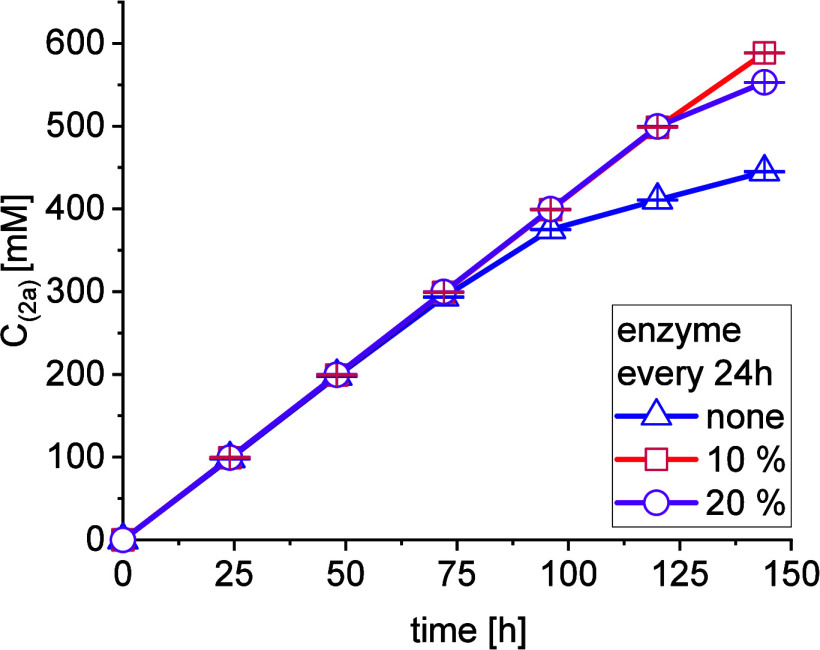
Time-dependent conversion for a 10 mL repetitive batch reaction of 100 mM **1a**, 120 mM **1b**, 5 mM PLP, 50 mM phosphate-buffer pH 8, 40 °C, 100 U/mL catalyst; additional enzyme was added after every 24 h cycle according to legend.

**Scheme 2:**
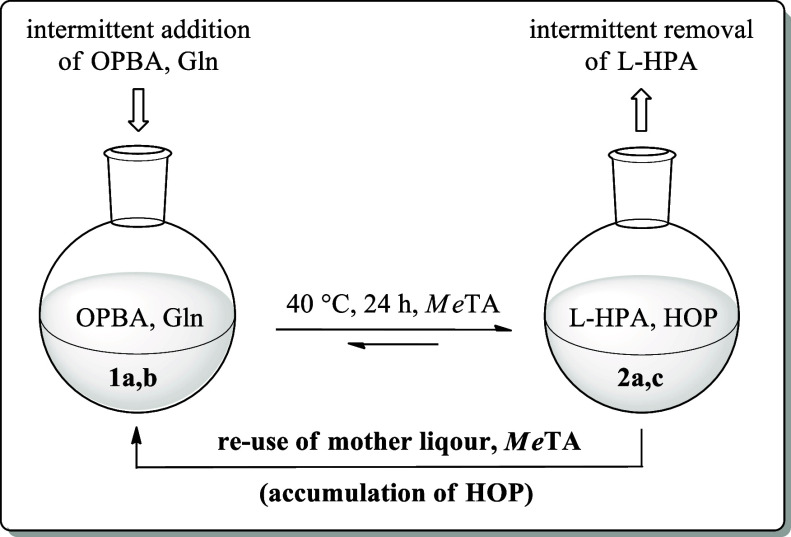
Process Concept of the Repetitive Batch Reaction with Re-Use of *Me*TA and Residual Reactants within the Remaining Mother Liquor for the Subsequent Cycle

After the initial screening, the reaction was scaled up to test its viability in a larger reaction volume, especially as the amount of the solid phase increased in parallel. This involved a 50 mL batch reaction, being mixed by a magnetic stirrer and heated to 40 °C, which resulted in a 98.7% conversion after 5 h, thus confirming the results seen at a smaller scale (see also Table 1 below). To further improve the overall concept of the reaction system, a repetitive batch concept was implemented (Scheme 2). This allows for reuse of the enzyme itself and more importantly the remaining mother liquor. After each reaction cycle, the crystallized product (**2a**) was separated from the reaction solution and the remaining liquid phase containing enzyme and PLP was then replenished with fresh substrates to continue the overall reaction. An additional enzyme was added to replace the inevitable loss of catalyst activity after 24 h (Figure 7). An exception to the overall trend is the sixth cycle, with 20% additional catalyst material after each cycle. This leads to a viscous reaction mixture due to catalyst accumulation after such a high cycle number, suggesting that catalyst overload needs to be avoided. The reaction system was found to be capable of almost complete conversion for 6 cycles, with 10% additional enzyme preparation after each cycle, even without the need for additional enzyme for the initial three reaction cycles, demonstrating high process stability of the reaction concept over multiple days. Additionally, the catalyst was not inhibited by the increasing concentration of coproduct **2c**, even at concentrations of >500 mM. This is important in allowing for the extraction of this secondary compound to be delayed, thus simplifying the down-streaming process.

**Table 1:** Comparison of Batch, Repetitive Batch, and Fed-Batch Reaction Using the *Me*TA-Catalyzed In Situ-Crystallization Concept with a Sequential Scale-Up Study^a^8

#	*V* [ml]	type	*X* [%]	STY [g/h·L]	*Z* [g/g]	isolated yield 2a [g]	isolated yield **2c** [g]
1	1	batch	99	3.6	0.6	n.d.	n.d.
2	50	Batch	99	3.6	0.6	n.d.	n.d.
3	6 × 10	Rep. B.	95	0.8	1.6	0.9	n.d
4	7 × 10	Rep. B.	98	1.6	3.2	1.1	0.5
5	340	Fed B.	95	2.4	6.2	18.5	9.6

**Figure 8:**
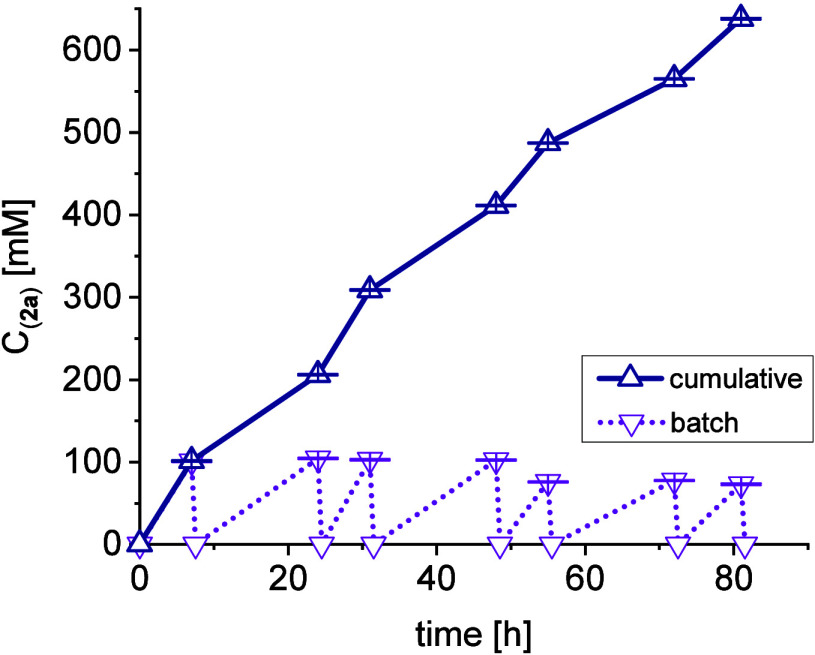
Time-dependent conversion for a 10 mL repetitive batch reaction of 100 mM **1a**, 120 mM **1b**, 5 mM PLP, 50 mM phosphate-buffer pH 8, 40 °C, 100 U/mL catalyst.

a X = conversion, STY = space time yield, *Z* = *m*_product_/*m*_enzyme_ = enzyme efficiency, n.d. = not determined, #3 = 24 h refill cycle, #4 = 8/16 h refill cycle (see Figures 7 and 8 for further details), experimental details are given in the respective chapters above.

The overall process was further optimized to fit the earlier shown full conversion after 5 h and transformed to an 8/16 h refill cycle procedure. The repetitive batch approach was successful in producing over 600 mM of product within 76 h (Figure 8, equals 1.1 g **2a**) when this shortened replenishment cycle was applied. In conclusion, a repetitive batch approach improves the utilization of the catalyst, which remains the most valuable part of this reaction. However, it results in a laborious synthesis that is difficult to scale up further. For this reason, the implementation of a fed-batch reactor setup was tested.

### Fed-Batch Reaction

**Scheme 3:**
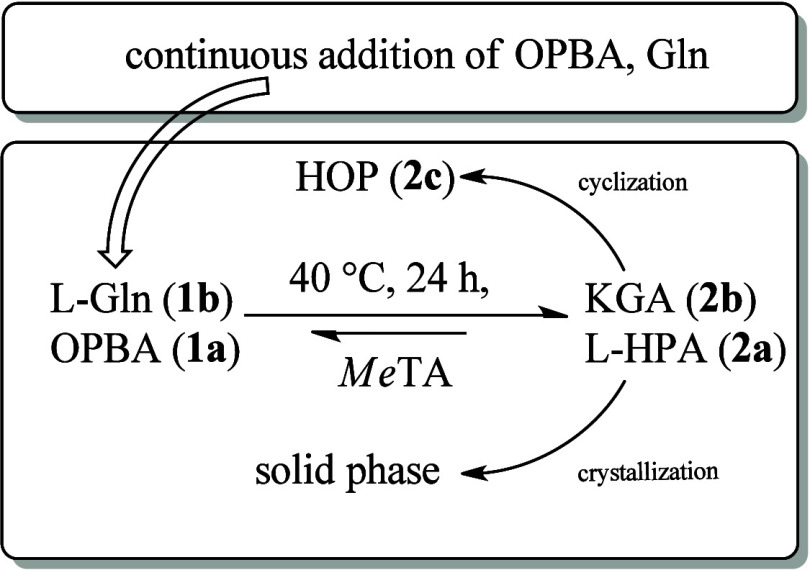
Process Concept of the *Me*TA-Catalyzed Fed Batch Reaction Over a 24 h Time Frame with a Continuous Feed of Fresh Substrates OPBA and Gln

Based on the repetitive batch experiments, the concept was changed to a fed-batch reaction system, as substrate inhibition of **1a** is the greatest challenge in this process. The setup was split into three main parts—a main reactor, a reservoir with dissolved substrates, and the pump—to continuously transfer these starting materials into the reactor. The initial conditions were set to 50 mM **1a** and 60 mM **1b** within a 500 mL flask (100 mL, 1 U/mL catalyst), into which a constant stream of 40 mM **1a** and 48 mM **1b** was added at a rate of 10 mL/h. This setup is intended to maintain a fast reaction velocity of the catalyst while keeping the concentration below 100 mM **1a** at all times, minimizing the risk of substrate inhibition. After a full 24 h reaction cycle, the synthesis was halted, a sample for HPLC analysis was taken, and both products were extracted from the reaction solution to be purified (Scheme 3). This concept allowed for the synthesis of 18.5 g **2a** with an enantiomeric excess of 99%. **2c** was extracted in parallel, yielding 10 g. Moreover, both products were tested for remaining protein impurities and the results showed <0.1% protein contamination (see the Supporting Information).

## Summary and Conclusions

Table 1 summarizes the overall conversion, space-time yield, and enzyme efficiency of the investigated coupled synthesis of both products, reactive crystallization of L-homophenylalanine, and adsorber-based downstream processing of 2-hydroxy-5-oxoproline. First, the classical batch reaction systems proved to be simple and optimal regarding simplicity, conversion, and space time yield, even when trialed at a larger scale (entries 1 and 2). However, enzyme efficiency was improved significantly when using repetitive batch or fed-batch reactor options as process alternatives. However, the STY initially decreased when converting a batch into a repetitive batch for two reasons (entries 3 and 4). First, there is a certain amount of downtime involved in refilling and restarting the reactor with fresh reactants. Second, due to constraints on working hours, the reactor was operated for a longer duration than necessary to reach complete conversion (24 h cycles vs 8/16 h cycles with higher productivity). This was a less significant constraint for the fed-batch process as it continuously fed fresh substrate into the biocatalytic synthesis reaction. This concept did not reach the same STY as a single batch reactor, as the substrate concentration was intentionally reduced to eliminate any possibility of substrate inhibition (entry 5, see also Figure 3). However, due to its simplicity, the presented final concept can be scaled up as required, only limited by the size of the reactor and the amount of catalyst that can be provided.

In summary, the gram-scale production and isolation of both compounds were easily achievable. HPA as a valued commodity product is an essential building block for the synthesis of active pharmaceutical ingredients (API), while the second product 2-hydroxy-5-oxoproline (**2c**) is even more valuable.

## Supporting Information Available

Additional experimental details, materials and methods, and NMR data (PDF)

## Abbreviations

Gln: l-glutamine **1b**
HOP: 2-hydroxy-5-oxoproline **2c**
HPA: homophenylalanine **2a**
aKGM: α-ketoglutaramate **2b**
*Me*TA: transaminase from *Megaphaera elsdenii*
OPBA: 2-oxo-4-phenylbutanoic acid **1a**
PLP: pyridoxal phosphate
