# Supplementary material for: Preparative Coupled Enzymatic Synthesis of L-Homophenylalanine and 2-Hydroxy-5-oxoproline with Direct In Situ Product Crystallization and Cyclization
Source: ACS Omega. 2025 Apr 2;10(14):14382–9. doi: 10.1021/acsomega.5c00590 (PMC12004150; doi:10.1021/acsomega.5c00590)
Supplement: Supplementary file 1 — ao5c00590_si_001.pdf [file ao5c00590_si_001.pdf]

Supporting Information:

Preparative Coupled Enzymatic Synthesis of L-Homophenylalanine and 2-Hydroxy-5-oxoproline with direct *in-situ*-Product Crystallization and Cyclization

Sven Tiedemann[a], Annabel Stang[a], Simon Last[a], Thierry Gefflaut[b], and Jan von Langermann[a]*

[a]Otto-von-Guericke University Magdeburg, Institute of Chemistry, Biocatalysis Group, Universitätsplatz 2, 39106 Magdeburg, Germany

[b]Université Clermont Auvergne, Institut de Chimie de Clermont-Ferrand, 24 avenue des Landais, 63178 Aubiere Cedex, France.

Table of contents

1. NMR Data from synthesized chiral amines	3
1.1 L-Homophenylalanine	3
1.2 2-oxo-5-hydroxyproline	4
2. NMR spectra	5
2.1: 1H-NMR of L-Homophenylalanine	5
2.2: 13C-NMR of L-Homophenylalanine	6
2.3: 1H-NMR of 2-oxo-5-hydroxyproline	7
2.4: 13C-NMR of 2-oxo-5-hydroxyproline	8
3. HPLC Chromatograms	9
3.1: Chromatograms of L-Homophenylalanine in comparison to enhanced rac. mixture	9
3.2: Chromatogram of purified 2-oxo-5-hydroxyproline	10
4. XRPD of L-Homophenylalanine	10
5. Preparation of biocatalyst	11
6. Protein Residue tests for products	11

1. NMR Data from synthesized chiral amines

^{1}H and ^{13}C-spectra were recorded on Bruker AVANCE 300WB and 600 spectrometers. The ee value is calculated by comparison of the LC-MS spectra via a racemic sample, which was prepared independently. All dry solvents were used as received.

1.1 L-Homophenylalanine

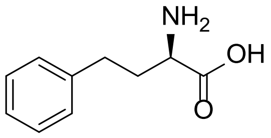

^{1}H-NMR (300 K, D_2O, 600 MHz) δ [ppm]: 2.25 (dm, J=52.02 Hz, 2H), 2.78 (m, 2H), 4.08 (t, 1H), 7.29 (m, 3H), 7.36 (2H)

^{13}C-NMR (300 K, D_2O, 150 MHz) δ [ppm]: 30.35 (s, 1C, Ph**C**H_2CH_2), 31.51 (s, 1C, $PhCH_2CH_2$), 52.36 (s, 1C, NH_2**C**COOH), 126.70 (s, 1C, **Ph**), 128.41 (s, 1C, **Ph**), 140.05 (s, 1C, Ph), 171.58 (s, 1C, NH_2C**C**OOH)

1.2 2-oxo-5-hydroxyproline

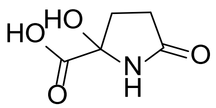

^{1}H-NMR (300 K, D_2O, 600 MHz) δ [ppm]: 2.15 (m, 1H), 2.42 (m, 1H), 2.55 (m, 2H)

^{13}C-NMR (300 K, D_2O, 150 MHz) δ [ppm]: 30.48 (s, 1C, NHCO**C**H_2CH_2), 34.40 (s, 1C, NHCOCH_2**C**H_2), 88.82 (s, 1C, NH**C**COOH), 175.39 (s, 1C, NH**C**OCH_2), 182.68 (s, 1C, NHC**C**OOH)

2. NMR spectra

All spectra were measured in $CDCl_3$ on a Bruckner 250 MHz NMR.

2.1: ^{1}H-NMR of L-Homophenylalanine

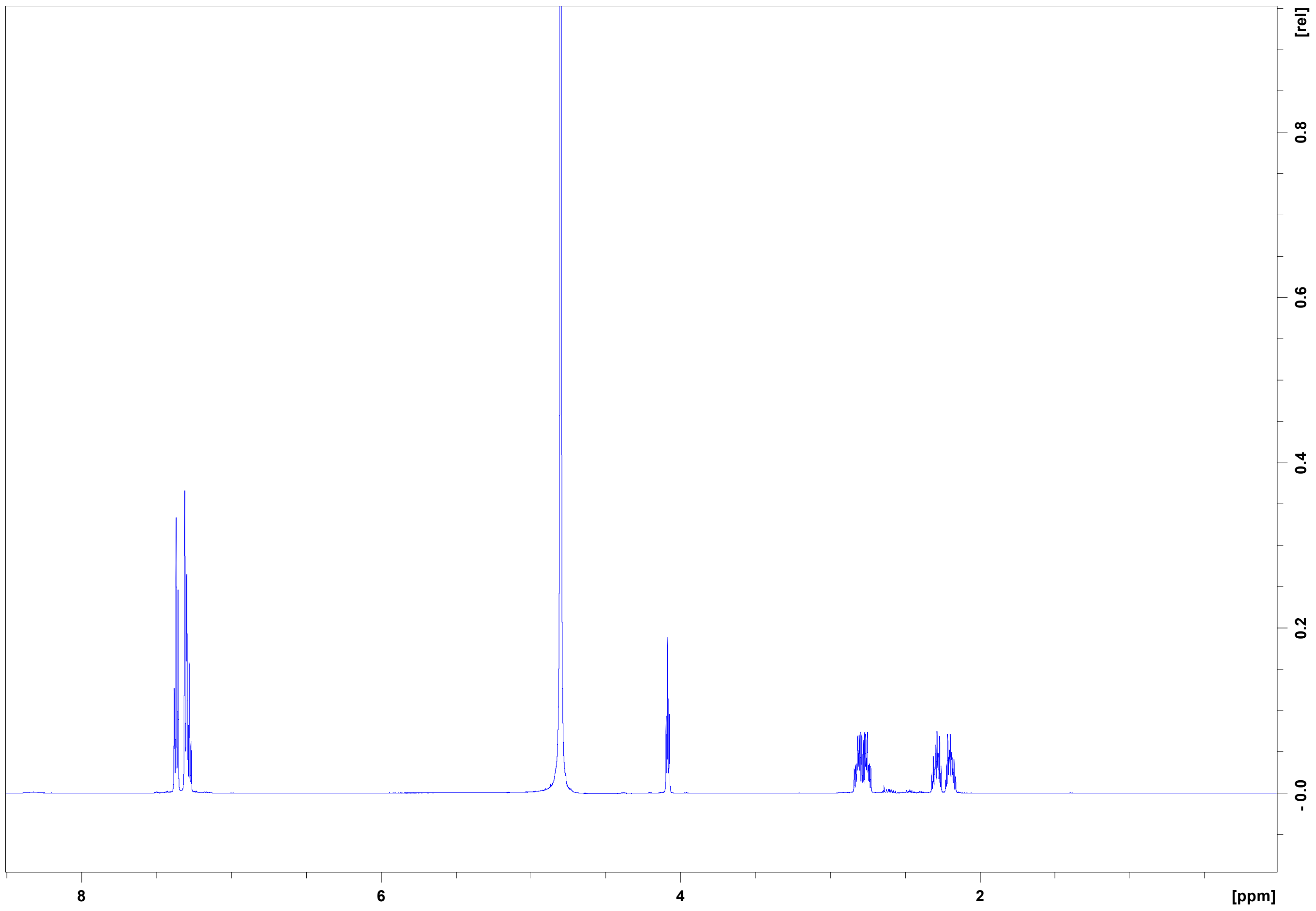

2.2: ^{13}C-NMR of L-Homophenylalanine

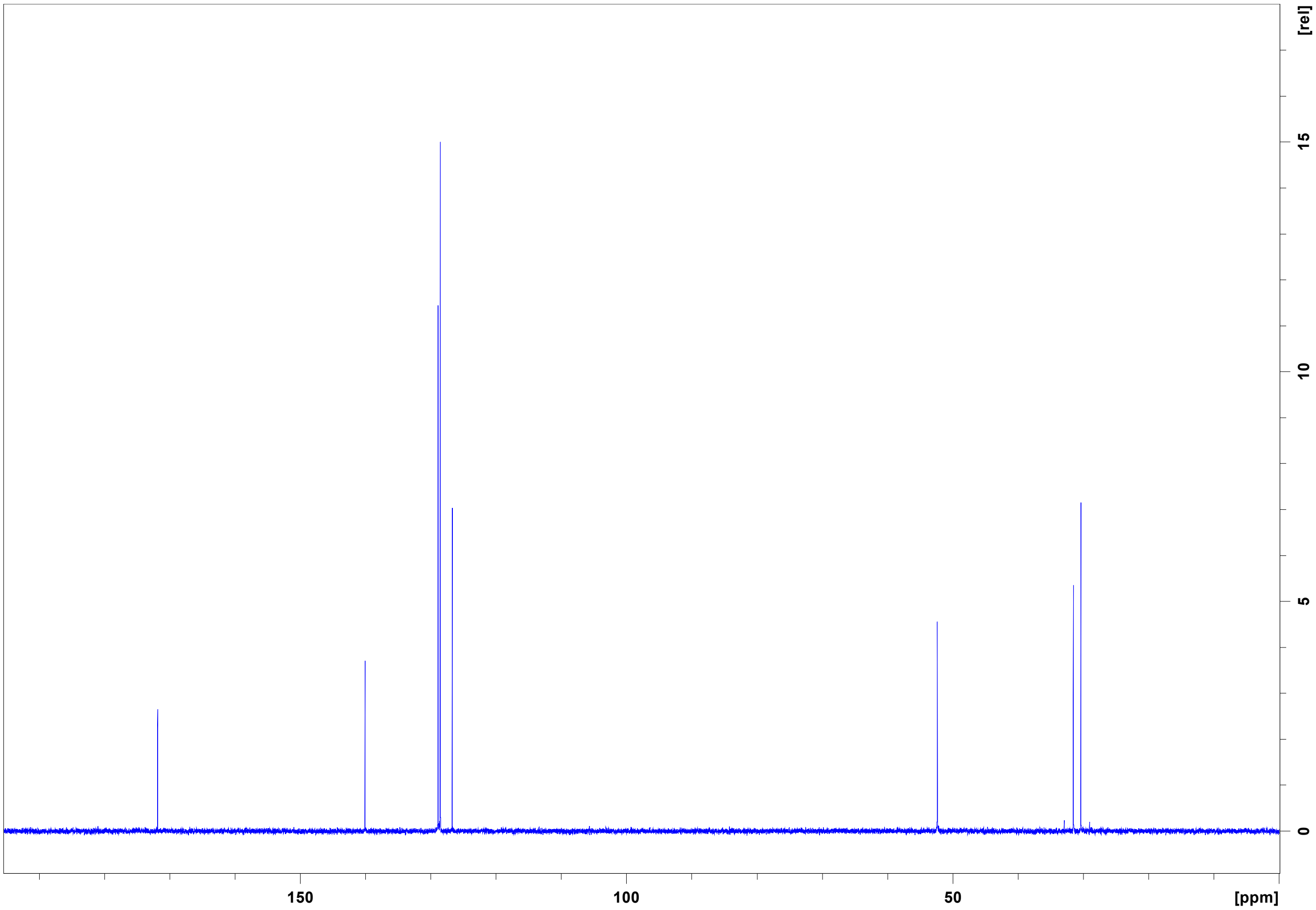

2.3: ^{1}H-NMR of 2-oxo-5-hydroxyproline

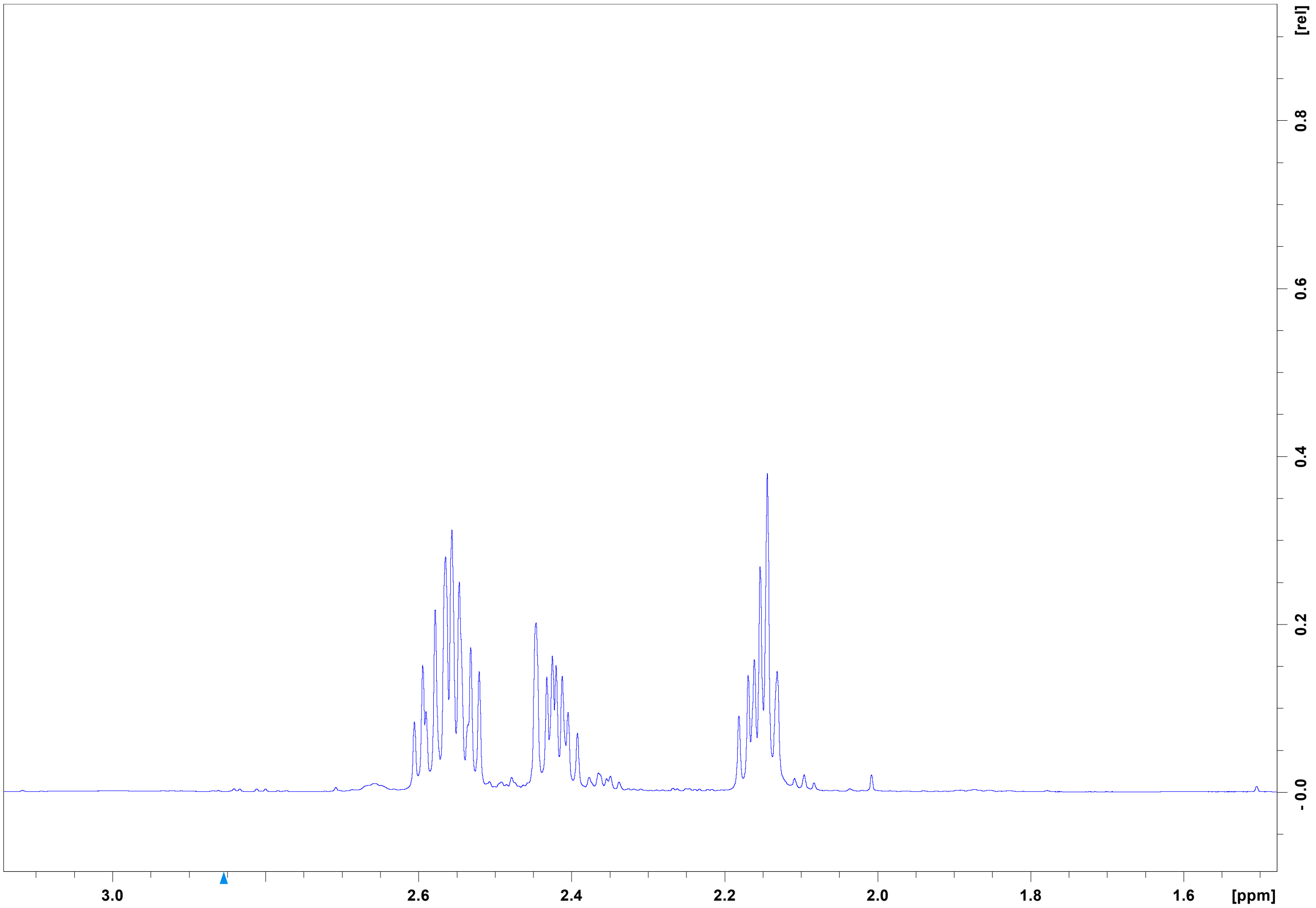

2.4: ^{13}C-NMR of 2-oxo-5-hydroxyproline

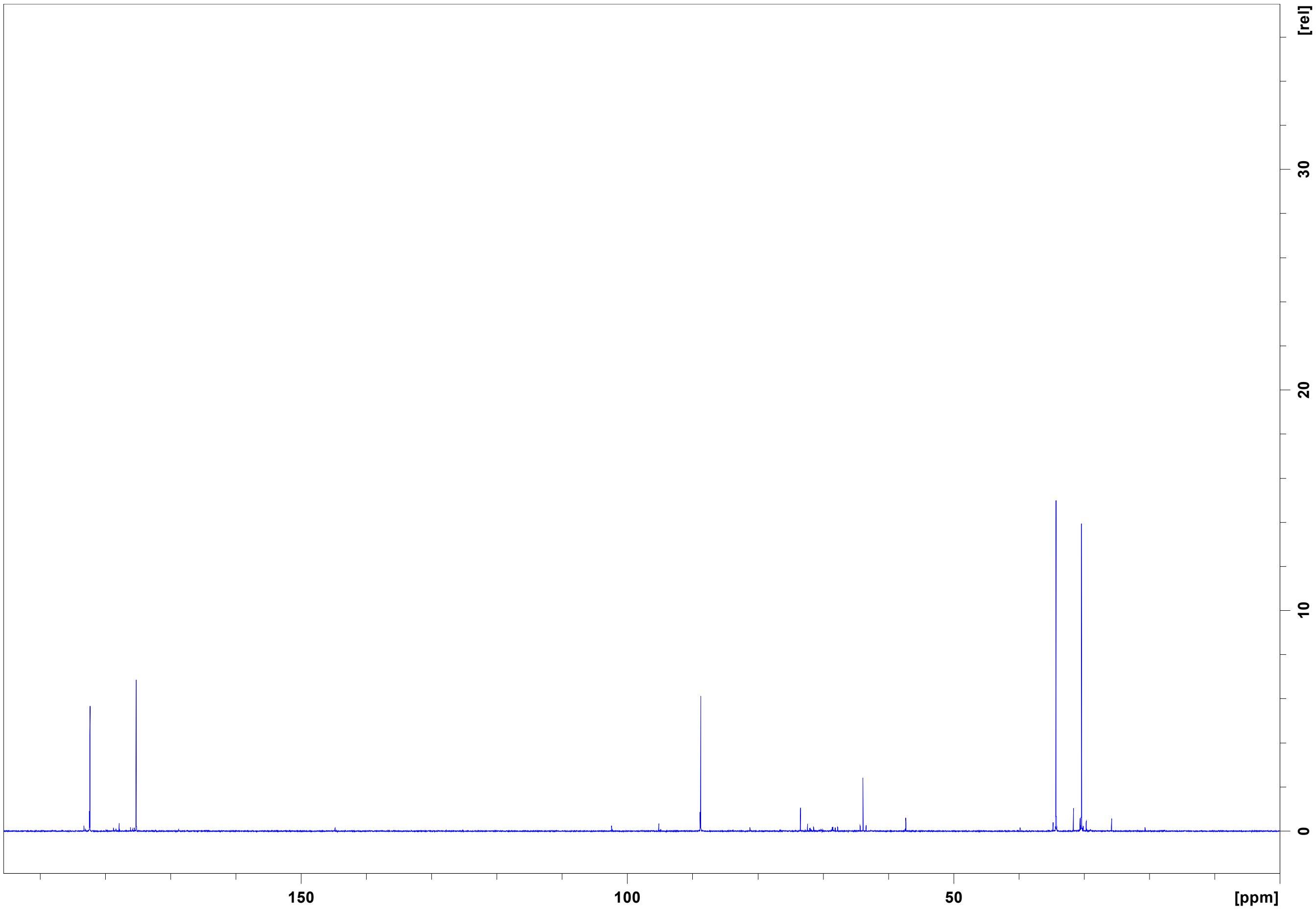

3. HPLC Chromatograms

All measurements shown are extracts from the corresponding LC-MS measurements at the respective product-specific mass. The *R* and *S* enantiomers were assigned by the reference measurements using commercially available chemicals.

3.1: Chromatograms of L-Homophenylalanine in comparison to enhanced racemic mixture

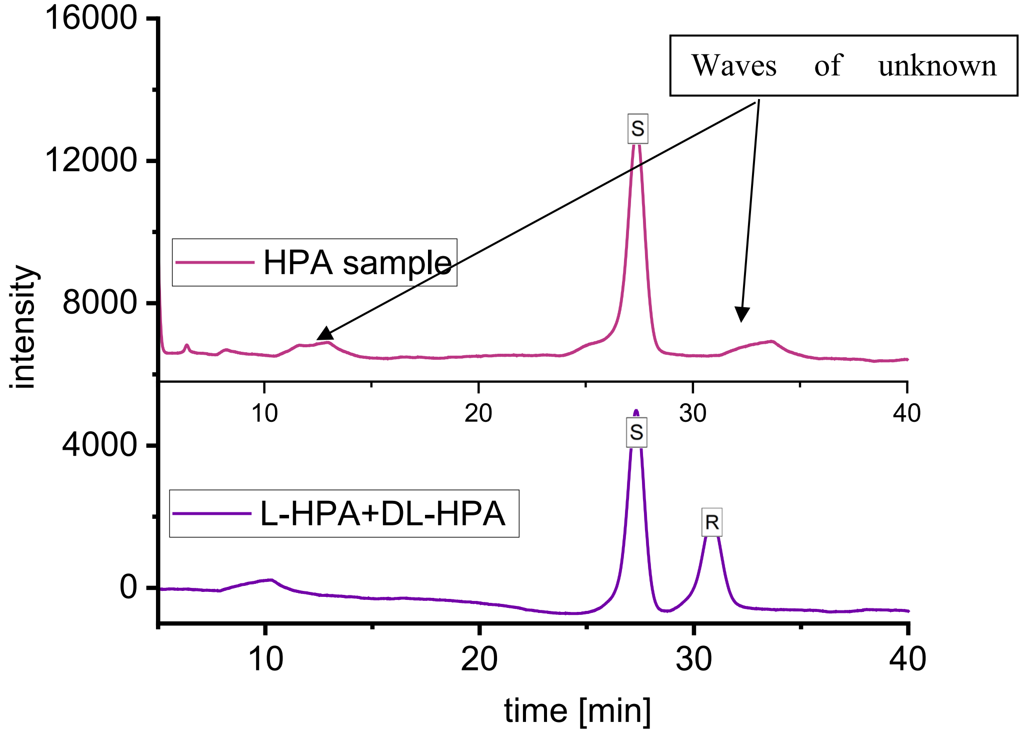

calculated ee: 99%

3.2: Chromatogram of purified 2-oxo-5-hydroxyproline

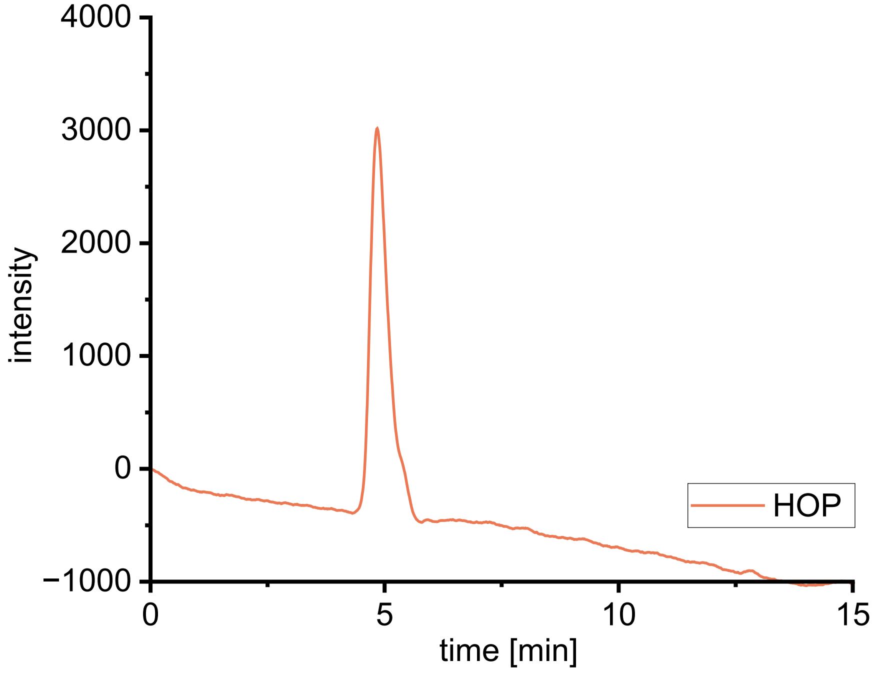

4. XRPD of L-Homophenylalanine

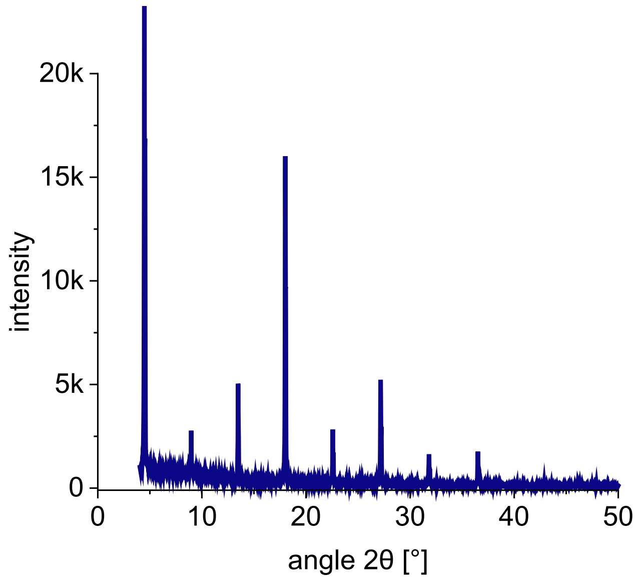

5. Preparation of biocatalyst

Amine transaminase from Silicibacter pomeroyi (SpATA); NCBI accession number: WP_011049154.1) was purchased from Enzymicals AG (Greifswald, Germany). For screening whole cells were used. In repetitive batch experiments cell free extract (recombinant in *E. coli*) were utilized. All samples existed in form of lyophilizates

6. Protein Residue tests for products

product	Calc. protein in [µg/mL test solution]	Calc. protein in [µg/g product]	Impurity in [w%]
L-HPA	3.9 ± 0.007	9.75 ± 0.02	<< 0.1 %
αKGA	1.5 ± 0.005	3.75 ± 0.01	<< 0.1 %

References

(1) Heuson, E.; Charmantray, F.; Petit, J.-L.; de Berardinis, V.; Gefflaut, T. Enantioselective Synthesis of d - and l -α-Amino Acids by Enzymatic Transamination Using Glutamine as Smart Amine Donor. Adv. Synth. Catal. 2019, 361 (4), 778–785.
(2) Nikulin, M.; Drobot, V.; Švedas, V.; Krasnikov, B. F. Preparative Biocatalytic Synthesis of α-Ketoglutaramate. International journal of molecular sciences 2021, 22 (23).
(3) Belov, F.; Mildner, A.; Knaus, T.; Mutti, F. G.; Langermann, J. von. Crystallization-based downstream processing of ω-transaminase- and amine dehydrogenase-catalyzed reactions. React. Chem. Eng. 2023, 8 (6), 1427–1439.
